# Theoretical framework and protocol for the evaluation of Strong Through Every Mile (STEM), a structured running program for survivors of intimate partner violence

**DOI:** 10.1186/s12889-019-6991-y

**Published:** 2019-06-04

**Authors:** Dayna M. Maniccia, Janel M. Leone

**Affiliations:** 1 School of Management, The Sage Colleges, 140 New Scotland Avenue, Albany, New York 12208 USA; 2 Department of Interdisciplinary Studies, The Sage Colleges, 140 New Scotland Avenue, 12208 Albany, New York USA

**Keywords:** Community-based intervention, Intimate partner violence, Physical health, Psychological health, Social support, Social well-being, Structured running program

## Abstract

**Background:**

Intimate partner violence can have a devastating impact on victims’ psychological and physical health and ability to maintain and preserve interpersonal relationships. The aim of the current study is to empirically test the effectiveness of Strong Through Every Mile (STEM), a 10-week structured running (exercise) program designed to increase psychological, social, and physical well-being among survivors of intimate partner violence. To the authors’ knowledge, STEM is the only community-based structured running program designed to improve the quality of life of survivors of intimate partner violence. This paper will describe the STEM program and present the theoretical basis of the program and the program evaluation design.

**Methods:**

The current study will utilize an interdisciplinary lens to evaluate a community-based intervention aimed at decreasing the negative effects of intimate partner violence on women’s lives. The study will use a mixed method approach (qualitative and quantitative), including a pre- and post-test evaluation of the STEM running program. Primary data will be collected using paper and pencil surveys which assess women’s psychological, social, and physical well-being prior to participation in the program and following the completion of the program. Qualitative data from focus groups will also be collected and allow for a more rich understanding of the changes that women experience over the course of the program and specific mechanisms underlying these changes.

**Discussion:**

The current study will employ an interdisciplinary lens to examine the extent to which a structured exercise program, specifically running, impacts the psychological, social and physical well-being of women survivors of intimate partner violence. Findings of this study can influence the development and implementation of similar programs for survivors of intimate partner violence and other types of trauma by identifying mechanisms central in achieving positive outcomes for participants.

## Background

Violence and trauma affect all aspects of a woman’s life including her physical and psychological health, self-sufficiency, employment, and ability to maintain social relationships [1]. While considerable scholarship has examined the prevalence and consequences of intimate partner violence against women, physical activity-based interventions designed to counter these consequences are sparse. To our knowledge, there are no published interventions that empirically test the effectiveness of a community-based structured running program as a mechanism to improve intimate partner violence survivors’ psychological, social, and physical well-being and/or their interpersonal relationships with their children or other family members.

Sport-for-development, using sporting activities to support opportunities beyond physical activity [2], is a promising mechanism to improve the health of individuals and communities. The current study will be the first to examine the impact of a structured running program on survivors of controlling intimate partner violence, intimate terrorism, which is characterized by physical violence embedded in a pattern of power and control. Compared to other types of intimate partner violence that may be more situationally-based, intimate terrorism is shown to have more immediate and long-term negative physical and psychological outcomes for victims and they are often forced to seek immediate intervention to escape or end the violence [3].

### Aim of the current study

This paper describes a “Couch-to-5 Kilometer” (C25K) running program developed specifically for survivors of intimate partner violence and the evaluation protocol that will be employed to assess program effectiveness. Such an evaluation is novel and theoretically informs our understanding of the complexities of survivors’ recovery and the ways in which community-based exercise programs can improve women’s overall well-being and interpersonal relationships. While STEM focuses directly on running and increasing physical fitness among participants, it is believed that the indirect benefits to participants span the spectrum of health. It is hypothesized, based on existing literature, that this program will have a positive effect on participants’ psychological, social, and physical well-being. This research project seeks to answer the following three research questions:

- Does participation in a structured running program positively impact the psychological health (e.g., self-esteem, self-confidence, happiness) of female survivors of intimate partner violence?
- Does participation in a structured running program positively impact the social well-being (e.g., perceived social support, relationships with friends and family members, social capital) of female survivors of intimate partner violence?
- Does participation in a structured running program positively impact the health behaviors (e.g., smoking, eating habits) and physical health (e.g., general health, physical pain, quality of life) of female survivors of intimate partner violence?

### Intimate partner violence

The World Health Organization defines health as “…a state of complete physical, mental and social well-being and not merely the absence of disease or infirmity” [4]. Intimate partner violence (IPV) against women is a public health issue [5] that impacts every aspect of health. IPV impacts 1 in 4 women in the United States [6] (almost 32 million women total [7]). In addition to the negative health impacts on women, IPV is associated with staggering economics costs ($103,767 per-victim over the course of the woman’s lifetime [7]).

#### Dynamics of intimate terrorism

Johnson and colleagues [3, 8–14] have empirically established that two major forms of male intimate partner violence against women exist. The distinction between the two forms - intimate terrorism and situational couple violence - is largely based around issues of power and coercive control. Intimate terrorism (IT) is defined as physical violence embedded in a broad pattern of power and control over one’s partner, where the violent partner exercises wide-ranging and pervasive coercive control meant to demonstrate power and ultimately entrap the partner in the relationship. In contrast, situational couple violence is physical violence against a partner that is situationally provoked, where one person may react physically to the pressures of a specific encounter or conflict. Research consistently finds that intimate terrorism is associated with more severe and frequent physical violence compared with situational couple violence [3, 12, 14]. However, the distinction between the two types is not based on physical violence; intimate terrorism is not a more severe “stage” of situational couple violence but rather a qualitatively different form of IPV.

Johnson’s largely control-based typology defines violence types on the basis of the underlying motivation to use physical violence, as demonstrated in a general framework of controlling behavior (e.g., isolation, threats, economic abuse). For an intimate terrorist, the control tactics serve to establish and demonstrate his own power while at the same time continuously weakening his partner’s power. For example, intimate terrorists entrap victims in the relationship by creating tremendous fear of further physical and sexual violence, by diminishing victims’ personal and financial resources, and by creating an environment for victims in which social support networks may be geographically and socially inaccessible [10]. Intimate terrorists actively work to deplete a victim’s power, thereby taking away any ability that she may have to stop the violence or escape the relationship. Intimate terrorists rely upon frequent and severe physical and sexual violence to emotionally and economically terrorize their victims. They often injure their victims, forcing them into crisis situations where urgent medical or legal intervention is necessary, even life saving [3, 10]. The population examined in the current study consists of survivors of intimate terrorism, women currently seeking shelter services to escape highly controlling physical and sexual violence perpetrated by a partner.

#### Impact of intimate terrorism on women

The negative consequences of highly-controlling intimate partner violence (i.e., intimate terrorism) on women’s well-being is well documented. In addition to violence-related injuries, there are psychological and physical health effects, as well as negative psychosocial and economic outcomes. The psychological effects of intimate terrorism include post-traumatic stress disorder (PTSD), anxiety, long- and short-term depression, lowered self-esteem, self-blame, and increased suicidality [12, 13, 15–17]. Socially, intimate terrorists often isolate their partners from friends and family members and victims often report a lack of perceived social support among friends and family members as well as lack of support from formal resources such as police and counselors [14, 18]. The negative impact of intimate partner violence on women’s ability to develop and maintain strong bonds with their children and with other family members has also been established (see Buchanan, Power, & Verity, 2014 [19] and Levendosky et al., 2004 [18]). Finally, non-injury-related physical health problems caused by intimate terrorism include sleep problems, headaches, chest pains, and gastrointestinal problems (see Campbell, 2002 [20] for a review).

#### Existing intervention strategies for intimate terrorism

Intervention strategies to counteract the harmful effects of highly-controlling intimate partner violence focus largely on providing immediate physical safety for women and their children (e.g., shelter programs, care for injuries, safety planning), and short- and long-term interventions to improve women’s psychological health (e.g., individual counseling, group counseling (see Sullivan, 2005 [21] for a review)). These programs typically involve victim advocacy, empowerment counseling, and cognitive behavioral therapy and have been shown to be psychologically beneficial [22]. Physical activity, however, has generally not been used to foster psychological, social, and physical well-being among this population. To date, the short- and long-term impact of such programs, including the one discussed in this paper, is unclear.

### Physical activity and well-being

Physical activity is positively associated with health-related quality of life [23–26]. Research has shown a positive link between running and various aspects of physical health including disease reduction and cardiovascular health [27–30]. Specifically, running is associated with improved aerobic fitness, resting cardiovascular functions, and metabolic fitness [29]. Meta analyses have found that running is associated with decreased body mass, body fat, resting heart rate, and triglycerides [28]. Running has also been positively linked to oxygen uptake (VO_2max_) and high density lipoprotein cholesterol [28].Additionally, physical activity is positively associated with psychological and social well-being among general and clinical populations, as well as trauma survivors, including intimate terrorism survivors. Among general populations of adults, physical activity has been significantly linked to increased psychological benefits [31, 32] including reductions in anxiety [33–38], reductions in depression [33, 37, 39–41], increased self-esteem [42, 43], improved ability to cope with stress [44], and improved life satisfaction [45]. Running specifically, has been linked to increased life satisfaction [46, 47]. Similarly, participation in sports - compared to overall physical activity such as that used as a means of transportation or for work - is associated with better psychological health [48]. Exercise has also been linked to increased positive body image, body esteem, and body satisfaction [49].

With regard to clinical populations of adults, physical activity is consistently and positively linked to psychological and social well-being [50]. Physical activity interventions for psychological health provide social interaction and support, a sense of meaning, purpose, and achievement, feelings of safety, and improved psychological symptoms [51]. Sports therapy, twice weekly participation in sports, for individuals receiving psychological health treatments is shown to increase feelings of accomplishment, well-being, self-esteem, positivity, alertness, positive mood, increased energy, and decreased listless [52]. Lastly, participation in physical activity by survivors of intimate terrorism is shown to improve resilience [53] as well as improve psychological and emotional status, including feelings of “normalcy”, and increase hopefulness, positive future outlook, and a sense of accomplishment [54]. Specifically, among individuals with post-traumatic stress disorder (PTSD), physical activity is negatively associated with depressive symptoms [55, 56] and PTSD symptoms [55, 57]. Additionally, among individuals with diagnosed depression exercise has a large antidepressant effect [58].

### Existing evaluations of community-based physical activity interventions

Despite strong evidence demonstrating the positive effects of physical activity on various components of well-being, few studies have specifically evaluated *structured running programs* to determine their impact on well-being. Several community-based running interventions exist for adolescents (e.g., *Students Run Philly Style* https://www.studentsrunphilly.org/; *Girls on the Run* http://www.girlsontherun.org; *The Just Run Program* http://www.justrun.org; *Ready Set Run* http://www.nays.org/programs/ready-set-run/; and *Kids Run the Nation* http://kidsrunthenation.org). To the authors’ knowledge, only *Girls on the Run* and *Students Run Philly Style* have been empirically evaluated. *Girls on the Run* is designed for pre-teen girls and combines training for a 5-km (5 K) (3.1 mile) run with a three-part curriculum that focuses on increasing self-awareness, self-care, team- and community-building, communication, and social engagement and awareness. Evaluation findings indicate that following the program, participants exhibit decreased body dissatisfaction and increased self-esteem [59]. Continued participation in the program is linked to continued improvement in self-esteem and body satisfaction [60]. *Students Run Philly Style* uses distance running to promote healthier living and high school completion among mostly minority and low socioeconomic status students aged 12 to 18. Students train to complete races of differing lengths including the Philadelphia Marathon or Half Marathon. Participation in the program is positively linked to motivation for running, general self-efficacy, attitude toward a healthy lifestyle, and engaging in higher levels of non-threatening behavior [61].

To the best of the authors’ knowledge, only one structured running program specifically focused on adults at-risk for psychosocial difficulties has been evaluated. *Back on My Feet (BoMF)* (http://www.backonmyfeet.org/) is a running program specifically for individuals experiencing homelessness. An evaluation of the *BoMF* program found that following program completion, participants reported increased self-sufficiency, self-esteem, self-confidence, and perceived productivity [62]. The current evaluation seeks to determine empirically if similar outcomes exist for survivors of intimate partner violence who participate in the STEM program.

## Methods/design

### Strong Through Every Mile (STEM) program design

Strong Through Every Mile (STEM) is a Couch-to-5 Kilometer (C25K) running program designed specifically for individuals receiving services through several domestic violence shelters in the Capital Region of New York (http://www.stemrunning.com/). A C25K running program is one that is designed for novice runners or those who are not consistent runners. Program participants alternate between running and walking during a 30-minute period. Over the course of several weeks, the duration of the running segment increases while the walking segment decreases until participants are entirely, or almost entirely, running. The STEM program, developed in 2013, consists of three (two week day and one weekend) organized group workouts per week for 10 weeks and culminates with a 5 K road race. The program is designed to build participants’ physical endurance and confidence, thus resulting in mastery experience.

STEM is a trauma-informed program that includes two sessions each year (Spring and Fall) and embraces an ecological framework for prevention [63, 64]. STEM promotes psychological, social, and physical health by encouraging healthy behaviors (individual level), specifically by promoting physical activity. STEM may contribute to self-esteem by filling one’s need for achievement and attention [65]. The social setting helps to build relationships and social capital (relationship/interpersonal level) while working with community-based organizations (community level). Training with individuals with similar life experiences facilitates adherence to the workout sessions as group cohesion encourages participation [66] and provides social support which positively impacts health [67]. Through fundraisers and events, STEM helps to create social awareness of intimate partner violence and post-traumatic personal growth (societal level).

The program was not developed to directly address past trauma, however there is an acknowledgment that prior traumatic experiences impact perceptions, behaviors, and thoughts – a central component of trauma informed approaches [68]. Trauma-informed approaches are grounded in the principles of physical and emotional safety, trust (of others and oneself), peer support, collaboration, empowerment [68, 69], and choice [69]. Trauma-informed practices acknowledge survivors have a need to be respected, become hopeful about their recovery [70], and connect with others [70, 71].

STEM is implemented and maintained entirely through volunteer efforts, and is funded solely through donations. To date, STEM has partnered with four local domestic violence shelters that provide residential and non-residential services for survivors of intimate partner violence. Each shelter has a designated staff member who serves as a STEM liaison/coordinator. The liaison works with STEM staff to explain the program to shelter clients. Interested individuals self-enroll in the program and are partnered with a “mentor”, a community volunteer who runs with participants during training sessions and the target race and provides support and encouragement.

Women must receive medical clearance prior to participating in STEM – shelter staff assist participants with receiving clearance if they do not have an established relationship with a medical provider. Once women are identified and medically cleared for participation, the liaison/coordinator works with STEM staff to schedule running sessions and identify a training location. The partner domestic violence shelters provide transportation to and from the training location and a shelter representative attends training sessions to provide support if needed. It is important to note that the women’s safety during training sessions is paramount. Research consistently shows that the escaping and help-seeking process can be extremely dangerous and involve a high degree of risk for victims entrapped by intimate terrorists. Indeed, escaping or trying to end the relationship is an immediate precipitating factor in 45% of cases in which men kill female partners [72]. Given that STEM participants are actively seeking services to end/escape the violence, they are particularly at-risk of being harmed or killed and measures are taken to protect women from such harm.

Initially, STEM provides participants with donated running shoes and athletic wear if necessary. Later, women who participate in the program for the first month are accompanied to a local running store and provided exercise clothing and a pair of new running shoes. A local athletic business has partnered with STEM to provide these supplies at a discounted rate. STEM provides team shirts for participants and pays the race registration fees for them. After the completion of the race, STEM participants gather for an end-of-session celebration.

#### STEM’s theoretical framework

The STEM program focuses on tertiary prevention, coping and recovery from intimate partner abuse [5]. STEM’s expected outcomes are supported by four main theoretical frameworks: self-determination theory, self-efficacy theory, locus of control, and social capital theory. Additionally, the empowerment, happiness, and mindfulness literature provides support for STEM’s anticipated outcomes.

##### Self-determination theory

Self-determination theory (SDT) explains human motivation and personality. SDT postulates that three key psychological needs (competence, relatedness, and autonomy) foster well-being [73]. Competence refers to the need to gain mastery of tasks, feel confident, and feel effective in task performance; relatedness or connection refers to the need to experience a sense of belonging, that one is cared for by others and, in turn, cares for others; lastly, autonomy refers to a need to feel in control of behaviors and goals. When these needs are met, growth, social development, and personal well-being are increased [74–76]. Satisfying these needs is necessary for optimal wellness and performance; failure to satisfy these needs results in negative psychological outcomes [74–76].

SDT proposes that these psychological needs are strongly associated with autonomous motivation [75]. Autonomous motivation describes behavior when individuals perceive themselves as having full choice, volition, interest, enjoyment, and value. When autonomously motivated, wellness, engagement, performance, and relationships are better. Self-direction, choice, and feelings of acknowledgement promote autonomous motivation [75]. In contrast, controlled motivation refers to doing something to gain a reward or avoid a punishment; performing due to feeling pressured or obliged to do so. Autonomous motivation is associated with increased persistence, greater vitality, and higher self-esteem, as well as general well-being compared to controlled motivation [75]. Autonomy orientation is associated with decreased negative impacts of stressful situations [73], greater psychological health [76], and increased general health [77]. Over the long term, fulfillment of the need for competence, relatedness, and autonomy leads to seeking out more situations that support these needs [73] thus supporting positive relationships and situations.

It is predicted that programs like STEM promote autonomous motivation by satisfying the basic needs described by self-determination theory (i.e., competence, relatedness, and autonomy) by offering a non-judgmental and voluntary framework which supports the development of autonomy orientation [73]. Participation in the STEM program is solely based on choice and there are little to no external rewards. Competence is gained by women gradually increasing their physical endurance (mastery experience) and thus confidence (self-efficacy) to successfully complete a 5 K road race. Mastering running techniques or even the ability to complete the course contributes to a sense of competence and increased confidence. The group format of the program provides a sense of connection and closeness, relatedness to others with similar life experiences. Survivors participate in the training and race together along with mentors and agency counselors; there is a sense of belonging and caring that grows over the course of the program. Lastly, autonomy is achieved through the program’s emphasis on non-judgemental support and participant self-direction. The program fosters behavior regulation based on one’s own goals, values, and interests rather than control orientation which involves coercion and pressure [73]. Participants are provided with a training plan but ultimately determine their own direction by choosing what they will do during each training session. Additionally, choosing to complete the 5 K is decided solely by the participant herself; she has complete and total control over the program’s completion.

##### Self-efficacy theory

Related to self-determination theory, self-efficacy theory is based in social learning theory [78] and focuses on the belief in one’s ability to accomplish a goal or task. Specifically, self-efficacy is the optimistic self-belief in competence and the prospect of successfully achieving or accomplishing a task. Mastery experience is one component of self-efficacy theory, and arguably the most powerful contributor to self-efficacy. Mastering a task or controlling an environment builds self-belief and provides the confidence to complete the same or similar task in the future. Self-efficacy is significantly impacted by participation in physical activity [79]. It is believed that the STEM program fosters a sense of mastery by offering participants the opportunity to train for and complete a 5 K race. The C25K format increases the run interval and decreases the walk interval in incremental fashion showing participants they can run for extended periods, gradually building on prior successes (a characteristic of mastery experience). Participants gain confidence in their ability to run. It is predicted that such confidence transfers to other aspect of their lives. While empowerment may be context specific, developing a sense of competence and control in one area of life may transfer to others as the actions to achieve goals are not as important as attempting to exert control [80].

##### Locus of control

Locus of control (LOC) [81] describes an individual’s belief system regarding the causes of, and factors that contribute to, successes and failures. An individual with a sense of personal control believes that he/she can master, control, and shape their destiny while individuals who perceive a lack of control believe that how they behave does not impact outcomes [82]. LOC is theorized to exist on a continuum, with several aspects that correspond to the extent to which individuals perceive outcomes in their lives as being within their control (internal locus of control) to being outside of their control (external locus of control). Perceptions of internal control are positively associated with empowerment [83], increased social action, lower psychological stress [80], and decreased depression [84].

Internal locus of control is central to survivors of intimate partner violence, particularly intimate terrorism, because of the nature of this type of violence. As discussed previously, intimate terrorists use economic control, intimidation, isolation, emotional abuse, and physical and sexual violence to gain and maintain control over their partner. Through entrapping a victim in the relationship, an intimate terrorist forces a sense of powerlessness upon the victim as he literally takes control of her life. Among survivors of intimate terrorism, trauma hinders perceptions of internal control and may subsequently lower perceptions of hope [85].

It is predicted that the STEM program will increase participants’ sense of accomplishment and internal control as participants are encouraged and empowered to set their own goals and create their own path to success. While the program determines the race, training schedule, and distance, the participants decide to join the program and control their training frequency and intensity. This type of control increases beliefs of personal control over one’s life and adoption of healthy behaviors [84].

##### Social capital theory

Social capital refers to the available resources, either actual or potential, resulting from one’s social network or ties between individuals [86] and the ability to secure benefits and resources from these networks [87]. Unlike physical capital (e.g., property, wealth) or cultural capital (e.g., education, skill set), social capital is a function of who we know, who is in our social circles, and the extent to which we engage with these people.

The theory of social capital suggests that one’s position within a particular group or social network provides certain benefits. Findings indicate a positive correlation between social capital and psychological health [67, 88]. Moreover, studies have demonstrated empirically that participating in group activities is linked to increased social capital. Ottesen and colleagues (2010) found that previously inactive women who participated in a 16-week physical activity program report bonding with individuals who were unlike themselves and had improved social relations with significant persons in their lives [89]. Findings also suggest that team sports are more effective at fostering social capital compared to physical activities performed alone or outside of a group [89] potentially due to the sense of community, which is central to social capital [86]. It is predicted that STEM will increase participants’ social capital through group interaction and the increased engagement with other program participants and mentors.

##### Mindfulness and happiness

Mindful individuals are aware of and pay attention to what is occurring in their internal and external environment [90] rather than performing in a habitual or automatic fashion [90, 91]. Mindfulness is related to increased relationship satisfaction [92], an improved ability to cope [90, 92, 93], increased well-being, and a positive view of stressful situations [93]. In addition to providing health benefits on its own, mindfulness promotes decision making that supports one’s own needs, values, and interests [90, 91]. Meta-analyses show that mindfulness, in addition to positively impacting psychological health, is related to increased subjective well-being [94] or happiness. Additionally, happiness is positively associated with physical activity [95–97] and physical health [98]. It is anticipated that participation in the STEM program will positively impact mindfulness and happiness.

### Evaluation methodology

#### Design, procedures and sampling

**Fig. 1:**
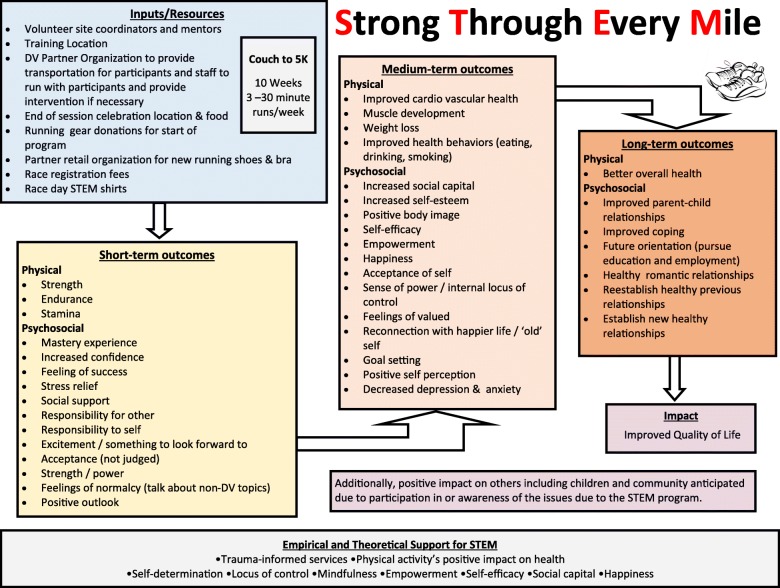
Logic Model of the Strong Through Every Mile (STEM) program. Logic model of the Strong Through Every Mile (STEM) program detailing required resources, anticipated short-, medium-, and long-term outcomes, overall impact on participants and theoretical and empirical basis supporting the program

The evaluation of the STEM program will be conducted in a two phases. In the first, the research team conducted focus groups with previous program participants, reviewed program material, and reviewed scientific literature to identify potential program outcomes and develop a theory- and data-driven program logic model (Fig. 1). The second phase of the study is the impact evaluation designed to assess program outcomes using a pre-test/post-test design. The evaluation aims to answer three main research questions: 1) Does participation in a structured running program positively impact the psychological health (e.g., self-esteem, self-confidence, happiness) of female survivors of intimate partner violence? 2) Does participation in a structured running program positively impact the social well-being (e.g., perceived social support, relationships with friends and family members, social capital) of female survivors of intimate partner violence? 3) Does participation in a structured running program positively impact the health behaviors (e.g., smoking, eating habits) and physical health (e.g., general health, physical pain, quality of life) of female survivors of intimate partner violence? The STEM program evaluation protocol was reviewed and approved by The Sage Colleges Institutional Review Board. All study participants will provide written informed consent prior to participation in the study.

#### Evaluation phase I – logic model and focus groups

Phase I of the program evaluation included development of the program logic model, review of program documents, and focus group data collection. Individuals unable to participate in focus groups but wishing to speak with researchers were interviewed in-person. All focus groups and interviews were recorded and transcribed by a research assistant. Transcripts were reviewed for themes related to participation in and examples of theoretical components of the program. All participants provided written informed consent.

#### Evaluation phase II – program effectiveness

The second phase of the evaluation is an impact evaluation to test hypothesized short- and medium-term outcomes. The goal is to collect data from all STEM participants with maximum efficiency and minimal intrusion of research staff in the STEM program or the partner agency service delivery. Principal investigators will coordinate pre- and post-test data collection times with partner agency staff. Individuals will complete the survey independently, or if requested, have the survey read to them by a member of the research team or partner organization. Participants will receive a running belt after completing the post-test survey. To protect confidentiality and to match pre-and post-test data, participants will be given a unique identification number. All participants will provide written informed consent prior to completion of the survey.

### Survey measures

#### Demographic information

Respondent relationship and sociodemographic characteristics will be recorded. These variables include age, race, education level, employment status, relationship status, length of relationship, and number of children.

#### Psychological well-being

Eight measures will be used to assess respondent psychological well-being: the 21-item Depression, Anxiety and Stress Scale (DASS 21) [99] assesses a range of depression and anxiety symptoms; the 12-item Self-Compassion Scale – Short Form [100] assesses self-kindness, self-judgement, isolation, and mindfulness; the 10-item Rosenberg Self-Esteem Scale [101, 102] measures global self-worth; the 50-item Self-Perception Profile for Adults Scale [103] measures perceptions of competence and adequacy; the 8-item Index of the Sense of Control [104, 105] assesses internal and external locus of control; the Mindful Attention Awareness Scale (MAAS) [91, 106] assesses mindfulness; the 8-item Brief Resilience Scale [107] assess resilience or “the ability to bounce back or recover from stress” (p.194); and the 4-item General Happiness Scale [108] measures subjective happiness.

#### Social well-being

Participants’ social well-being will be measured by the 14-item Interpersonal Support Evaluation List (ISEL) [109] which assesses perceived social support and social capital. Participants’ interactions with their children will be assessed with the 8-item Parental Warmth/Acceptance scale from the parents’ version of the Child’s Report of Parental Behavior Inventory (CRPBI) [110–112].

#### Physical well-being

The World Health Organization Quality of Life instrument [113] will be used to assess respondent physical health. This measure includes 26 items which measure broad domains of physical health, psychological health, social relationships, and environment. Selected questions from the Behavioral Risk Factor Surveillance System Questionnaire [114] will assess quality of life and changes in health behaviors.

#### Future behavior

Future behavior will be assessed by asking participants about their behavioral intentions. Intention is a reliable predictor of behavior [115, 116], thus providing the ability to assess whether STEM has impacted participants’ long term planning as it relates to exercise, employment, and education.

#### Sample size calculations

The target sample size for this study was calculated using G*Power [117]. To calculate an a priori sample size, a commonly accepted alpha level of 0.05, and power level of 0.80 as recommended by Cohen [118] were use. An effect size of 0.4, one in the middle of the moderate range (0.3 - 0.5) was selected for use in sample size calculations. (Standard conventions classify an effect size of 0.2 as small, 0.5 as moderate, and 0.8 as large [118]). Sample size calculations using these parameters estimated a sample size of 54 is needed to detect significant change if it exists.

#### Data analysis

SPSS will be used to conduct descriptive and other analyses. Chi-squared or t-test analyses will be used to compare baseline and post-test samples. Participant pre-test data will be compared to post-test data using a paired T-test. Sensitivity analyses will be conducted to assess robustness of the results.

## Discussion

The intervention being studied incorporates multiple psychological and sociological theories including self-determination theory, self-efficacy theory, locus of control, social capital, empowerment, happiness, and mindfulness. The STEM running program engages community members as mentors, collaborates with local domestic abuse assistance agencies, and elicits financial support for running apparel from area businesses, including a local running specialty store. Through community engagement, the program has the unintended consequence of raising awareness of the issue of intimate partner violence and survivors’ ability to thrive.

The current study seeks to provide a multifaceted, empirically-derived understanding of the impact of a structured running program on survivors of intimate terrorism. The program evaluation will inform future research directions and program expansion and delivery. The interdisciplinary nature of this study establishes a dynamic collaboration between the fields of family science, psychology, and public health and bridges an often ambiguous gap between these disciplines. A future step will be to determine STEM’s effectiveness compared to other common interventions including non-physical activity based programs such as support groups, book clubs, and sewing circles. Additionally, long term studies can examine the physiological markers of stress experienced by survivors of intimate partner violence over the course of the STEM program. The incorporation of physiological data into this work will help elucidate the underlying mechanisms responsible for STEM’s beneficial effect on participants.

The findings have important implications for informing and influencing practice regarding community responses to survivors of violence and ultimately improving the provision of services for survivors. If the current study, and future multi-group studies, demonstrate STEM’s effectiveness, robust evidence will exist for a unique, low-cost community-based intervention to promote survivor well-being.

## Abbreviations

5 K: five kilometer
BoMF: Back on My Feet
C25K: Couch-to-5 K
CRPBI: Child’s Report of Parental Behavior Inventory
DASS21: 21 item Depression Anxiety and Stress Scale
IPV: intimate partner violence
ISEL: Interpersonal Support Evaluation List
LOC: locus of control
MAAS: Mindful Attention Awareness Scale
PTSD: Post-traumatic stress disorder
STD: Self-Determination Theory
STEM: Strong Through Every Mile
WHO: World Health Organization
